# Doula Care and Health Outcomes: A Systematic Review

**DOI:** 10.1001/jamanetworkopen.2026.8416

**Published:** 2026-04-21

**Authors:** Peyton Groves, Hannah Williams, Cynthia L. Salter, Abigail Smith Kosbie, Adena Bowden, Ruben G. Martinez, Jessica Davis, Dara D. Méndez, Maya I. Ragavan

**Affiliations:** 1 Division of General Academic Pediatrics, University of Pittsburgh and UPMC Children’s Hospital of Pittsburgh, Pittsburgh, Pennsylvania; 2 Department of Behavioral and Community Health Sciences, University of Pittsburgh School of Public Health, Pittsburgh, Pennsylvania; 3 Healthy Start Incorporated, Pittsburgh, Pennsylvania; 4 Department of Psychiatry and Human Behavior, The Warren Alpert Medical School, Brown University, Providence, Rhode Island; 5 Department of Epidemiology, University of Pittsburgh School of Public Health, Pittsburgh, Pennsylvania

## Abstract

**Importance:**

Maternal-infant health inequities remain a critical public health issue in the US, where adverse maternal and infant health outcomes disproportionately affect American Indian or Alaska Native and Black populations. Doula care—where trained doula professionals provide physical, emotional, and informational support across the perinatal continuum—is a community-based strategy to improve outcomes.

**Objective:**

To systematically evaluate clinical and pilot trial evidence on the association of doula care with maternal and infant health outcomes; identify gaps in study design, equity, and implementation; and propose future directions for clinical practice, health systems research, and policy.

**Evidence Review:**

This systematic review included articles published between January 1, 2000, and January 31, 2026. Searches in PubMed, PsycINFO, Web of Science, and CINAHL, plus reference and expert review, identified eligible clinical or pilot trials assessing maternal and infant outcomes. Protocols, secondary analyses, abstracts, and non-English articles were excluded. Data on study design, intervention characteristics, outcomes, and risk of bias were qualitatively synthesized.

**Findings:**

Twenty-two articles describing 21 unique interventions met inclusion criteria. Most were randomized clinical trials (17 of 21 [81.0%]), with others using quasiexperimental or preference-based designs. Interventions had heterogeneity in scope, intensity, and doula training. Findings were most consistent for improved maternal anxiety, postpartum follow-up, and breastfeeding initiation, while evidence for outcomes such as cesarean delivery and pain management was more variable. Few studies addressed postpartum, abortion, or broader reproductive health contexts (eg, fertility or bereavement doulas). Methodologic gaps included limited reporting of fidelity and implementation, underrepresentation of marginalized populations, and failure to account for crossover in control groups.

**Conclusions and Relevance:**

The findings of this systematic review of clinical trials suggest that doula care may improve perinatal anxiety, health care utilization, and breastfeeding initiation, although the evidence for other outcomes was inconsistent. Future studies should use rigorous designs, ensure equitable representation, examine full-spectrum care, and assess implementation to support sustainable integration into maternal health systems.

## Key Points

**Question:**

What evidence from clinical trials supports the association of doula care with maternal and infant health outcomes?

**Findings:**

In this systematic review of 22 articles describing 21 unique studies, doula care was most consistently associated with improved maternal anxiety and breastfeeding initiation and emerging evidence on improved postpartum follow-up. Results across other outcomes were heterogeneous, with interpretation limited by variable study design, underrepresentation of marginalized populations, and poor reporting of intervention fidelity.

**Meaning:**

These findings suggest that doula care shows promise but requires more rigorous, equity-focused research to clarify its effectiveness across diverse settings.

## Teaser

This systematic review assesses the evidence base for understanding how doula programs can equitably and sustainably improve maternal-child health and highlights opportunities to strengthen the role of doulas through research, policy, and practice.

## Introduction

Maternal-infant health inequities remain a critical public health crisis in the US. Certain racial and ethnic groups experience higher rates of cesarean delivery,^1^ preterm birth,^2^ and severe morbidity,^3^ persisting even after adjusting for comorbidities^4^ and educational attainment.^5^ These inequities are driven by structural and social determinants of health, including the chronic stress of systemic racism^6,7^; addressing them requires interventions beyond routine health care.

Doula care, which is often community-based and culturally concordant, represents one such strategy. A doula is a trained professional who provides physical, emotional, and informational support across the perinatal experience.^8^ Services include education and hands-on techniques to enhance comfort, confidence, and emotional health. Demand for doula care is increasing in the US,^9^ alongside expanding Medicaid coverage^10,11^ and endorsement by the American College of Obstetricians and Gynecologists.^12^ Existing studies that have evaluated doula support delivered at different time points across the perinatal period, including prenatal, intrapartum, and postpartum care, have demonstrated positive health outcomes such as lower rates of cesarean delivery,^13,14,15^ higher rates of breastfeeding initiation,^16^ and improved mental health outcomes.^14,17^ However, heterogeneity in study design and rigor limits causal inference across broader health outcomes.

To address this gap, we conducted a systematic review of clinical trials worldwide evaluating doula care and its effects on maternal and infant health outcomes. Doula care was defined broadly as services and support provided by trained doulas across the perinatal period and in other situations where doulas could be used (eg, fertility support). Doula care can take place in various settings, including community-, home-, and hospital-based care. Clinical trials provide the strongest evidence for causality, allowing us to build on prior observational research with greater rigor. Evaluating study quality, scope of doula care, and implementation is key to understanding how doula programs can equitably and sustainably improve maternal-child health. This review aims to critically assess the evidence base and highlight opportunities to strengthen doulas’ role through research, policy, and practice.

## Methods

### Eligibility Criteria

The protocol was registered in PROSPERO (CRD42024617663). The University of Pittsburgh Institutional Review Board deemed the study non–human participant research and therefore waived the need for ethics review and informed consent. We followed the Preferred Reporting Items for Systematic Reviews and Meta-Analysis (PRISMA) reporting guideline. Studies were eligible if they were (1) original research on health impacts of doula care, broadly defined as support provided by trained doulas in any setting across care contexts, not limited to the perinatal period; (2) clinical or pilot trials; (3) published between January 1, 2000, and January 31, 2026 (to focus review on up-to-date, relevant studies); and (4) published in English. Secondary analyses, protocol papers, conference abstracts, and dissertations were excluded to ensure studies in the sample reported on original data and outcomes.

### Data Sources and Search Strategy

An electronic literature search was conducted in November 2024 using PubMed, PsycINFO, Web of Science, and CINAHL (Figure). Search terms included *doula* or *birth worker* or *community health worker* and *health*. Of 2047 unique records, 137 underwent full-text review after title and abstract screening. Two reviewers (P.G. and H.W.) independently assessed eligibility, resolving discrepancies by consensus. Sixteen articles met all eligibility criteria. Reference review and expert consultation identified 2 and 3 additional eligible studies, respectively. To ensure completeness, a repeat search was conducted for publications from December 1, 2024, to January 31, 2026, identifying 1 additional eligible study (Figure).

**Figure.**
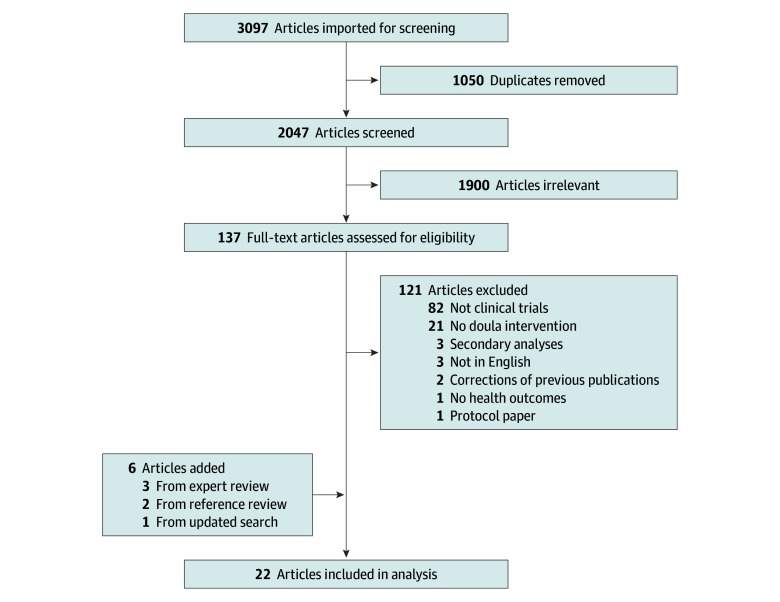
Flow Diagram of Study Selection for the Identification of Peer-Reviewed Publications on Impacts of Doula Interventions

### Data Extraction

One researcher (H.W.) abstracted study data, verified by 2 others (P.G. and M.I.R.). A data abstraction codebook was developed for (1) study descriptions and included interventions; (2) study design; and (3) intervention evaluation. Two reviewers (P.G. and H.W.) independently assessed evidence quality using the Cochrane RoB-2 (risk of bias assessment tool 2), ROBINS-I (risk of bias in nonrandomized studies of interventions), and Oxford Centre guidelines (Table 1 and eTable in Supplement 1).

**Table 1.** Risk of Bias Assessment

Assessment tool and outcome	Studies
RoB-2 score for RCTs	
Low concerns	Mottl-Santiago et al,^18^ 2008; Burris et al,^19^ 2025
Some concerns	Akbarzadeh et al,^20^ 2015; Campbell et al,^21^ 2006; Chor et al,^22^ 2015; Edwards et al,^23^ 2013; Hans et al,^24^ 2013; Hans et al,^25^ 2018; Luo et al,^26^ 2025; Masoudi et al,^27^ 2022; McGrath and Kennell,^28^ 2008; Schytt et al,^29^ 2022; Wilson et al,^30^ 2017; Zhang et al,^31^ 2020; Trueba et al,^32^ 2000
High concerns	De Moraes et al,^33^ 2024; Gjerdingen et al,^34^ 2013; Ravangard et al,^35^ 2017
ROBINS-I score for non-RCTs	
Low risk of bias	Zhang et al,^36^ 2018
Serious risk of bias	Shahbazi Sighaldeh et al,^37^ 2023
Critical risk of bias	Chen and Lee,^38^ 2020; Gruber et al,^39^ 2013

Abbreviations: RoB-2, Cochrane risk of bias assessment tool 2; ROBBINS-I, risk of bias in nonrandomized studies of interventions.

## Results 

### Intervention Topics, Settings, and Doula Involvement 

In total, 22 articles^18,19,20,21,22,23,24,25,26,27,28,29,30,31,32,33,34,35,36,37,38,39^ on 21 unique interventions were included in this review (Table 2). Two articles^23,24^ derived from the same primary study reported on separate primary outcomes. Two of the 21 studies (9.5%) were pilot trials.^33,34^ All 21 studies took place during the perinatal period; 12 (57.1%) focused on the intrapartum period,^20,21,26,27,28,31,32,33,35,36,37,38^ 2 (9.5%) focused on the postpartum period only,^19,34^ 5 (23.8%) spanned prenatal through postpartum care,^18,23,24,25,29,39^ and 2 (9.5%) occurred during abortion procedures.^22,30^ Six studies (28.6%) targeted low-income communities,^18,19,21,23,24,25,39^ and 6 (28.6%) included nulliparous individuals.^18,28,29,32,33,35^ Ten studies (47.6%) did not specify a target population.^20,22,26,27,30,31,34,36,37,38^

**Table 2.** Study Descriptions and Included Interventions

Study	Intervention topic	Setting	Target population	Type of doula support	Duration of doula support	Approach	Doula training
Akbarzadeh et al,^20^ 2015	Compare effects of doula care and acupressure on the mother’s anxiety level and delivery outcome	Hospital	Pregnant individuals seeking childbirth	Birthing	Throughout labor and delivery	Individual	Not specified
Campbell et al,^21^ 2006	Compare labor outcomes in women accompanied by a doula vs women without an additional support person	Hospital	Low-income pregnant women	Birthing	Throughout labor and delivery	Individual	DONA International certified; 4 h additional training on traditional doula topics
Chen and Lee,^38^ 2020	Compare outcomes in women accompanied by a doula vs women without an additional support person	Hospital	Pregnant individuals seeking childbirth	Birthing	Throughout labor and delivery	Individual	DONA International certified
Chor et al,^22^ 2015	Evaluate impact of doula support on first-trimester surgical abortion care	Hospital	Pregnant individuals receiving first trimester surgical abortion care	Surgical abortion	Throughout procedure and in recovery area	Team: 3 doulas provided support during the study period, 1 experienced and 2 who completed abortion doula training and subsequent proctoring	6 Lectures, group discussions, and role playing, including medical and psychosocial aspects of abortion care, pregnancy options counseling, clinic logistics, and team-building topics
de Moraes et al,^33^ 2024	Evaluate whether the continuous support provided by doulas influences the endogenous release of serotonin in parturient	Hospital	Healthy nulliparous pregnant women	Birthing	Throughout labor and delivery	Individual	Not specified
Edwards et al,^23^ 2013	Examine effects of a community doula home visiting intervention on infant feeding practices	Hospital and community	Low-income Black mothers	Prenatal, birthing, and post partum	Home visits from pregnancy through 3 mo post partum and support during childbirth	Individual	20-wk Doula training course and 10-wk breastfeeding peer counselor training program provided by the Chicago Health Connection
Gjerdingen et al,^34^ 2013	Evaluate impact of postpartum doula and peer telephone support as adjunctive treatments for depressive symptoms	Community	New mothers experiencing postpartum depression	Post partum	24 h of Study-sponsored postpartum doula services by trained, certified doulas offered for 6 wk	Individual	DONA International and CAPPA certifications
Gruber et al,^39^ 2013	Compare birth outcomes between a group of mothers with doula support and increased education vs those without	Hospital and community	Socially disadvantaged mothers	Prenatal, birthing, and post partum	2 Prenatal visits, continuous assistance throughout labor and birth, and ≥2 postpartum visits	Individual	DONA International certified
Hans et al,^24^ 2013	Examine efficacy of a community doula intervention in supporting behavioral, attitudinal, and emotional aspects of the early parent-child relationship	Hospital and community	Young mothers (aged <22 y) residing in low-income areas	Prenatal, birthing, and post partum	Prenatal home visitation, support during labor and delivery, and 3 mo of postpartum home visitation	Individual	10-wk Training session provided by the Chicago Health Connection
Hans et al,^25^ 2018	Examine impact of doula home visiting on birth outcomes, postpartum maternal and infant health, and newborn care practices	Hospital and community	Young low-income families	Prenatal, birthing, and post partum	Prenatal home visitation, support during labor and delivery, and 6 wk of postpartum home visitation	Team: participants assigned a home visitor (also called a family support worker or parent educator) and a community doula	Not specified
Luo et al,^26^ 2025	Evaluate efficacy of integrating nursing early warning systems with doula-assisted childbirth nursing on natural childbirth rates and associated outcomes	Hospital	Pregnant individuals seeking childbirth care	Birthing	Labor and delivery as well as first hours directly after birth	Team: participants assigned a doula and a health educator	Not specified
Masoudi et al,^27^ 2022	Assess effect of labor supportive care and acupressure on mother’s anxiety level and arterial oxygen pressure of the umbilical cord	Hospital	Pregnant individuals seeking childbirth care	Birthing	Labor and delivery as well as first hours directly after birth	Individual	Not specified
McGrath and Kennell,^28^ 2008	Examine perinatal effects of doula support during labor and delivery	Hospital	Middle- and upper- income nulliparous women accompanied by a male partner throughout labor and delivery	Birthing	Throughout labor and delivery	Individual	DONA International certified
Mottl-Santiago et al,^18^ 2023	Evaluate the effectiveness of doula support in reducing rates of CDs and preterm births, as well as on breastfeeding outcomes	Hospital and community	Nulliparous, lower-risk pregnant people with public insurance coverage	Prenatal, birthing, and post partum	2-h Prenatal home visits (range, 1-8); continuous support through labor and birth; and 2-h visits (range, 1-4) through 6-8 weeks post partum	Individual	12 h Additional training on social determinants of health resources
Schytt et al,^29^ 2022	Evaluate effectiveness of community-based bilingual doula support for improving the intrapartum care experiences and postnatal well-being of migrant women	Hospital and community	Nulliparous women aged ≥18 y who do not speak fluent Swedish	Prenatal, birthing, and post partum	≤2 Prenatal visits; continuous support through labor and birth; ≤2 postpartum visits	Individual	8-d Training on physiology of childbirth, strategies for providing effective continuous support in labor, breastfeeding, and communication with clinicians
Shahbazi Sighaldeh et al,^37^ 2023	Compare maternal outcomes in the care provided by doula, trained lay companion, and routine midwifery care in the labor and obstetric units	Hospital	Pregnant individuals seeking childbirth care	Birthing	From active phase of labor through 1 h after birth	Individual	Not specified
Wilson et al,^30^ 2017	Evaluate impact of doulas on patients’ physical and emotional responses to surgical management of a first-trimester failed or undesired pregnancy	Hospital	Pregnant individuals seeking surgical health care related to terminating a pregnancy or treating a miscarriage	Surgical abortion	Throughout procedure and in recovery area	Individual	2-d Training in full-spectrum doula care from the registered nurse of the Penn Family Planning and Pregnancy Loss Center
Zhang et al,^31^ 2020	Evaluate whether a multifaceted intervention would decrease the CD rate in China	Hospital	Pregnant individuals seeking childbirth care	Birthing	Throughout labor and delivery	Team: due to a high volume of deliveries, a doula is often shared by >1 laboring woman	Not specified
Zhang et al,^36^ 2018	Investigate the effects of pain relief during labor on the occurrence of potential postpartum depression in early postpartum	Hospital	Pregnant individuals seeking childbirth care	Birthing	Throughout labor and delivery	Individual	Not specified
Ravangard et al,^35^ 2017	Assess anxiety and pain level of women giving birth using physiologic methods (without doula support) during labor and those women supported by a doula	Hospital	Nulliparous pregnant women seeking childbirth care	Birthing	Throughout labor and delivery	Individual	Not specified
Trueba et al,^32^ 2000	Evaluate whether doula support to women in labor decreases the possibility for surgical birth	Hospital	Nulliparous pregnant women seeking childbirth care	Birthing	Throughout labor and delivery	Individual	Doula training seminar at Lamaze International Childbirth Educator program at Anahuac University
Burris et al,^19^ 2025	Determine whether embedding doulas and CNMs for postpartum care in the NICU would reduce the time to receive postpartum health care	Hospital, NICU	Postpartum parents of infants who were born at GA <34 wk, <2 wk, and anticipated to remain in NICU ≥1 wk	Post partum	Duration of hospitalization	Individual	Not specified

Abbreviations: CAPPA, Childbirth and Postpartum Professional Association; CD, cesarean delivery; CNM, certified nursing-midwife; NICU, neonatal intensive care unit.

Most research studies (15 [71.4%]) were hospital based,^19,20,21,22,26,27,28,30,31,32,33,35,36,37,38^ meaning doula care occurred exclusively while the patient was in the health care setting. One study (4.8%) was implemented in exclusively community settings,^34^ while 5 studies (23.8%) included both hospital and community components.^18,23,24,25,29,39^ Geographically, more than half of the studies (11 [52.4%]) occurred in the US,^18,19,21,22,23,24,25,28,30,34,39^ 4 studies (19.0%) occurred in Iran,^20,27,35,37^ 3 studies (14.3%) occurred in China,^26,31,36^ and 1 study each occurred in Taiwan,^38^ Mexico,^32^ Sweden,^29^ and Brazil.^33^

#### Description of Doula Care

Seventeen studies (81.0%) used an individualized doula model^18,19,20,21,23,24,26,27,28,29,30,32,33,34,35,36,37,38,39^ and 4 (19.0%) used a team-based approach.^22,25,26,31^ Team-based interventions involved home visitors,^25^ doula trainees,^22^ or health educators.^26^ One team approach assigned multiple patients per doula given limited doula availability.^31^ One study matched doulas to participants based on race or ethnicity,^39^ and another based on language.^29^

Regarding doula training, 5 articles (23.8%) described researcher-led training tailored to study objectives,^18,22,23,24,29,30^ including social determinants of health,^18^ pregnancy loss,^22,30^ breastfeeding,^23,24^ or communication skills.^29^ Seven articles (33.3%) used a standardized training course such as certification from DONA International,^21,24,28,32,34,38,39^ and 10 studies (47.6%) did not specify the training method.^19,20,25,26,27,31,33,35,36,37^

The frequency and intensity of doula care ranged from continuous support during labor (12 studies^20,21,26,27,28,31,32,33,35,36,37,38^ [57.1%]) to multiple structured prenatal or postpartum visits (6 studies^18,23,24,25,29,34,39^ [28.6%]). In studies of labor support, doulas provided motivational coaching, repositioning, and advocacy. Prenatal and postpartum support centered on emotional counseling, preparation for labor, and guidance on infant care and self-care. Three studies reported specific time offered by doulas, such as 24 hours during a 6-week period^34^ or as a minimum number of prenatal and postpartum visits, which ranged from 1 to 8 visits in each period.^18,39^ Only 1 study reported on the extent of participant engagement, finding that participants had a mean of 10 prenatal and 10 postpartum visits.^24^

### Study Design 

#### Trial Types

Seventeen of 21 studies (81.0%) were randomized clinical trials.^18,19,20,21,22,23,24,25,26,27,28,29,30,31,32,33,34,35^ The remaining 4 studies (19.0%) used nonrandomized trial designs, including participant preference allocation^36,39^ and quasiexperimental designs.^37,38^ No studies were described as hybrid effectiveness-implementation trials. Study design is summarized in Table 3.

**Table 3.** Study Designs

Study	Country	Randomization model	Sample size and calculation	Inclusion and exclusion criteria	Recruitment setting	Control group	Third arm or additional intervention
Akbarzadeh et al,^20^ 2015	Iran	Stratified block randomization	150; Sample size calculated on anxiety	First or second pregnancy, singleton, aged 18-35 y, without contraindications to labor; smoking and tobacco use excluded	On presentation to hospital for delivery	Routine care	Acupressure at the BL32 point as third arm
Campbell et al,^21^ 2006	US	Simple randomization	586; Sample size calculated on CD rate	Nulliparous, low-risk, singleton pregnancy without contraindications to labor	Women’s ambulatory care center located at a tertiary perinatal care hospital	Routine care	NA
Chen and Lee,^38^ 2020	Taiwan	Quasiexperimental; did not elaborate further	220; Sample size calculation not provided	No pregnancy complications, including premature birth or history of fetal or neonatal death	Clinical outpatient medical center	Routine care	NA
Chor et al,^22^ 2015	US	Block randomization	214; Sample size calculated on pain	First-trimester abortion, English speaking, aged ≥18 y	Women seeking a first-trimester surgical abortion	Routine care	NA
de Moraes et al,^33^ 2024	Brazil	Block randomization	24; Sample size calculation not provided; pilot study	Nulliparous, singleton, term pregnancies without comorbidities, including those that interfere with the endogenous release of serotonin such as pancreatic diseases and enteropathies	On presentation to hospital for delivery	Routine care	NA
Edwards et al,^23^ 2013	US	Block randomization	248; Sample size calculation not provided	Women at <34 wk in pregnancy, aged <21 y, planning to keep their infant and live locally	Community health center and prenatal clinic	Routine care	NA
Gjerdingen et al,^34^ 2013	US	Simple randomization	39; Sample size calculation not provided; pilot study	Women with a positive PHQ-9 score (≥10), English speaking, aged ≥16 y, with infant aged 0-6 mo	3 St Paul, Minnesota, hospitals, local practices, mothering Web sites, and early childhood and family education	All participants were mailed a PPD brochure and a PPD resource list	Peer telephone supporters as third arm
Gruber et al,^39^ 2013	US	Nonrandomized; group selection based on participant preference	226; Sample size calculation not provided	Expectant mothers who attended ≥3 YWCA Healthy Moms Healthy Babies childbirth classes	Education classes at YWCA	Routine	NA
Hans et al,^24^ 2013	US	Block randomization	248; Sample size calculation not provided	Aged ≤22 y; ≤34 wk in pregnancy; planning to keep their infant and live locally	2 Affiliated prenatal clinics: one in a community health center and another in a nearby teaching hospital	Routine prenatal health care and social services offered through the clinics	NA
Hans et al,^25^ 2018	US	Stratified randomization (by community)	312; Sample size calculation not provided	Inclusion: aged <26 y, GA <34 wk, residing in the catchment area, and meeting HFA/PAT risk criteria; exclusion: aged <14 y, child welfare or justice involvement, or cognitive impairment	Not specified	Control group received case management services	Additional family support worker or parent educator in addition to doula care
Luo et al,^26^ 2025	China	Simple randomization	150; Sample size calculated on CD rate	Aged ≥20 y; term, singleton pregnancy without contraindications to labor	On presentation to hospital for delivery	Routine care	Music therapy and early nursing warning signs in addition to doula care
Masoudi et al,^27^ 2022	Iran	Block randomization	150; Sample size calculated on anxiety	Aged 18-35 y; term singleton pregnancy in spontaneous labor; having no history of clinical, mental, or surgical problems	On presentation to hospital for delivery	Routine care	Acupressure at the BL32 point as third arm
McGrath and Kennell,^28^ 2008	US	Simple randomization	420; Sample size calculated on CD rate	Nulliparous women aged 18-41 y in the third trimester of uncomplicated pregnancy, accompanied by male partners	Enrolled at childbirth education classes	Routine care	NA
Mottl-Santiago et al,^18^ 2023	US	Block randomization	367; Sample size calculated on CD rate	Nulliparous, singleton pregnancy; aged ≥18 y, GA 16-24 wk, without high-risk obstetric comorbidities	Not specified	Routine care; 25 control group participants accessed doula care	Medical Legal Partnership Boston services in addition to doula care
Schytt et al,^29^ 2022	Sweden	Block randomization	164; Sample size calculated on patient satisfaction and PPD scores	Women at GA 25-35 wk who were Arabic, Polish, Russian, Somali, or Tigrinya speaking and could not communicate fluently in Swedish, aged ≥18 y, had no contraindications for vaginal birth	Information about study provided during usual antenatal care visits between GA 25-35 wk	Routine care; telephone interpretation offered	NA
Shahbazi Sighaldeh et al,^37^ 2023	Iran	Quasiexperimental design: random sampling used, but no random group allocation due to small sample	150; sample size calculation on labor length and maternal anxiety	Inclusion: low-risk pregnant women aged 18-40 y, GA 38-42 wk, spontaneous onset of labor pains, and healthy, live, single fetus with display of head; exclusion: previous CD or LBW	Not specified	Routine midwife care	Trained lay companion as third arm
Wilson et al,^30^ 2017	US	Block randomization	70; Sample size calculated on pain scores	Inclusion: aged ≥18 y, presenting with a first-trimester failed or unwanted pregnancy, who desired office uterine evacuation for management of their pregnancy; exclusion: pregnant as a result of sexual assault, non-English speaking	On presentation to Penn Family Planning and Pregnancy Loss Center	Routine care	NA
Zhang et al,^31^ 2020	China	Stratified cluster randomization (by hospital)	21 273; Sample size determined by proportionate random sampling (10%-20% of births per hospital), not by a priori power calculation	Inclusion: low-risk pregnancies defined as cephalic presentation, mothers aged 18-40 y, term birth (37-41 wk), prepregnancy BMI 17- 28, and without previous ART, CD, prior or current stillbirth, and comorbidities contraindicating labor	Not specified	Routine care	Targeted patient health education on CD and new hospital CD policy, in addition to doula care
Zhang et al,^36^ 2018	China	Nonrandomized; group selection based on participant preference	565; Sample size calculation on PPD	Inclusion: aged 20-35 y, literate, completed full-term pregnancy (37-42 wk), a low-risk pregnancy, no history of smoking and alcohol abuse, and no history of depression; exclusion: psychiatric comorbidities, previous need for emergency intervention	Convenience sampling on presentation to hospital	In this study, doulas were considered the control group	Transcutaneous nerve stimulation and epidural use were the other 2 arms
Ravangard et al,^35^ 2017	Iran	Simple randomization	150; Sample size calculation not provided	Nulliparous Iranian women aged 16-44 y without any pregnancy complications or comorbidities related to thyroid, kidney, heart, liver, diabetes, or psychologic disorders	On presentation to hospital for delivery	Routine care	NA
Trueba et al,^32^ 2000	Mexico	Simple randomization	100; Sample size calculation not provided	Nulliparous women without a history of CD; term pregnancies in spontaneous labor	On presentation to hospital for delivery	Routine care	NA
Burris et al,^19^ 2025	US	Block randomization	37; Sample size calculation on time to first postpartum visit and blood pressure check, contraception counseling, and PPD screening	Inclusion: postpartum parents of NICU infants born at GA <34 wk, aged <2 wk; exclusion: not able to provide informed consent in English or for whom the medical team anticipated infant death or transfer to another hospital within 1 wk	EHR screening and contact via telephone or in-person for enrollment	Routine care	NA

Abbreviations: ART, assisted reproductive technology; BMI, body mass index (calculated as weight in kilograms divided by height in meters squared); CD, cesarean delivery; EHR, electronic health record; GA, gestational age; HFA/PAT, Health Families America/Parents as Teachers; LBW, low birth weight; NA, not applicable; NICU, neonatal intensive care unit; PHQ-9, 9-item Patient Health Questionnaire; PPD, postpartum depression.

#### Recruitment and Follow-Up

Fourteen of 18 pregnancy-focused studies (77.8%) excluded patients with high-risk conditions,^18,20,21,25,26,27,28,31,32,33,35,36,37,38^ including maternal diabetes, extremes of maternal age, and any condition contraindicating labor. Fifteen studies (71.4%) measured outcomes within 24 hours of the birth.^18,20,21,22,23,26,27,29,30,31,32,33,35,37,38,39^ The remaining 6 studies reported follow-up at 3 to 4 weeks,^36^ 6 weeks,^28^ 12 weeks,^19^ 3 to 4 months,^25^ 6 months,^34^ and 24 months.^24^

#### Control Groups and Additional Interventions

Seventeen studies (81.0%) compared doula care with an unspecified standard care.^18,19,20,21,22,23,24,26,27,28,30,31,32,33,35,37,38,39^ Three studies described an active control group, including case management,^25^ educational materials on postpartum depression (PPD),^34^ or language interpretation.^29^ One study in China used the doula group as the control, comparing it with 2 interventions: epidural and transcutaneous electrical nerve stimulation.^36^

Five studies (23.84%) included a third arm, such as acupressure,^20,27^ telephone peer support,^34^ a trained lay companion,^37^ and transcutaneous nerve stimulation.^36^ In another 4 studies (19.0%), doula care included additional enhancements, such as music therapy,^26^ a family support worker,^25^ legal services,^18^ and a multifaceted approach combining enhanced maternal education with policy changes to reduce cesarean delivery rates.^31^ Only 1 study reported whether the control group accessed doula care^18^: 25 of the 180 control participants accessed doula care but remained analyzed as controls since they did not receive the study’s legal services.

### Intervention Evaluation

#### Demographic Characteristics

Results of interventions are summarized in Table 4. Mean ages across studies ranged from 18 to 31 years. Four studies (23.8%) primarily enrolled Black patients^19,22,23,24,39^; 1 study (4.8%) focused on Hispanic populations,^18^ 1 study (4.8%) enrolled a population that was mostly White,^28^ and remaining studies included mixed populations.^20,21,25,26,27,29,30,31,32,33,34,35,36,37,38^ In international studies, participant race and ethnicity and language generally reflected national demographic characteristics, except 1 study in Sweden that used bilingual doulas interpreting for refugee mothers.^29^

**Table 4.** Study Results

Source	Participant characteristics	Outcomes assessed	Timing of outcome assessment	Measurement tools	Implementation outcomes assessed
Akbarzadeh et al,^20^ 2015	30% Aged 21-25 y (majority); mean GA, 38.9 (range, 37-41 wk); 74.7% homemakers; 43% middle-school completion only	Maternal anxiety (decreased),^a^^,^^b^ labor length (decreased),^b^ CD (decreased)^b^	Beginning of active phase of labor and end of first stage of labor	State-Trait Anxiety Inventory	NA
Campbell et al,^21^ 2006	Mean age, 22 y (both groups); age range, 14-40 y; race and ethnicity, 29%-36% Black, 18%-21% Hispanic, 56% White	CD,^a^ oxytocin use, epidural rate, labor length (decreased),^b^ 1- and 5-min Apgar score (increased),^b^ cm dilated at epidural (increased)^b^	Immediately after birth	NA; medical record review only	NA
Chen and Lee,^38^ 2020	Educational range, junior high school through PhD; 30% unemployed in doula group and 32% in control group; age and ethnicity not reported	CD (decreased),^b^ labor length (decreased), oxytocin (increased, thought due to higher natural birth rate),^b^ analgesics, forceps delivery, meconium, maternal anxiety, maternal depression, pain, Apgar scores, epidural rate	Beginning of active phase of labor and immediately after birth	State-Trait Anxiety Inventory; Edinburgh Postnatal Depression Scale; Pain Visual Analog Scale; Mackey Childbirth Satisfaction Rating Scale	Patient satisfaction
Chor et al,^22^ 2015	Mean age, 24 y (both groups); race and ethnicity, 85% Black in doula group and 90% in control group; marital status, 87% single in doula group and 91% in control group	Pain,^a^ procedure duration, maternal anxiety	Before, during (speculum insertion), and immediately after procedure	Pain Visual Analog Scale	Patient satisfaction
de Moraes et al,^33^ 2024	Mean age, 23 y in doula group and 21 y in control group; marital status single, 58% in doula group and 41% in control group	Serotonin concentration (increased),^b^ CD, birth weight, Apgar scores, labor length, oxytocin use, analgesics, episiotomy, perineal injury	Active phase, expulsion phase, and immediately after birth	NA: medical record review only	Intervention fidelity
Edwards et al,^23^ 2013	Mean age, 18 y (both groups); race and ethnicity, 100% Black; mean GA, 23 wk; 88.7% nulliparous; 93.8% Medicaid coverage	Solid food introduction (closer to 6 mo),^b^ breastfeeding initiation (increased),^b^ breastfeeding duration	Immediately after birth, the second morning after birth, and 4 mo post partum	Self-reported breastfeeding	NA
Gjerdingen et al,^34^ 2013	Mean (SD) age, 29.7 (5.7) y; race and ethnicity, 95% White; marital status, 84% married; 87% employed during pregnancy	Maternal depression and prepregnancy depression (increased, attributed to baseline differences),^b^ general maternal health state (increased),^b^ general infant health state, missed workdays	0, 3, and 6 mo Post partum	PHQ-9; Center for Epidemiologic Studies–Depression Scale; 5-item Available Support Survey; 7-item Importance of Support Survey	Patient satisfaction
Gruber et al,^39^ 2013	Mean age, 19 y in control group and 20 y in doula group; age range, 13-31 y; race and ethnicity, 78% Black, 6%-8% White, and 15% other	Breastfeeding initiation (increased),^b^ LBW (decreased),^b^ CD, birth complications	Immediately after birth	NA: medical record review only	NA
Hans et al,^24^ 2013	Mean age, 18 y (both groups); race and ethnicity, 100% Black; mean GA, 23 wk; 88.7% nulliparous; 93.8% Medicaid coverage	Sensitive parenting attitudes (increased),^b^ sensitive parenting practices (increased),^b^ positive parent-child interactions (increased),^b^ parental stress (decreased at 12 mo only)^b^	4, 12, and 24 mo post partum	Parent-Child Observation Guide; Mother Encouragement and Guidance Scale	NA
Hans et al,^25^ 2018	Mean age, 18 y (both groups); race and ethnicity, 45% Black, 38% Hispanic; marital status single, 29%; mean GA, 25 wk	Safe sleep practices (increased),^b^ safe car seat practices (increased),^b^ breastfeeding initiation (increased),^b^ analgesics (decreased),^b^ epidural (decreased),^b^ maternal and infant rehospitalization, maternal depression, pediatric follow-up, preterm birth, LBW, CD	Pregnancy and 3 wk and 3 mo post partum	Center for Epidemiologic Studies–Depression Scale	NA
Luo et al,^26^ 2025	Ages 17-40 y; mean (SD) age, 28.5 (4.3) y in control group vs 29.1 (4.0) in doula group	CD (decreased),^a^^,^^b^ postpartum hemorrhage (decreased),^b^ maternal anxiety (decreased),^b^ pain (decreased), 1- and 5-min Apgar score (increased),^b^ labor length (decreased)^b^	During labor and immediately after birth	Visual Pain Analog Scale	Doula and patient satisfaction (increased)
Masoudi et al,^27^ 2022	Employment, 74.7% homemaker; educational level, 43.3% primary school completion only; mean (SD) GA, 38.92 (1.17) wk	Maternal anxiety (decreased),^a^^,^^b^ umbilical cord oxygen (increased)^b^	Beginning of active phase of labor and end of first stage of labor	State-Trait Anxiety Inventory	NA
McGrath and Kennell,^28^ 2008	Mean age, 28 y; race and ethnicity, 78% White; marital status, 88% married; educational level, 57% college completion; 100% middle-upper income	CD (decreased),^a^^,^^b^ epidural (decreased)^b^	24 h and 6 wk Post partum	Medical Record Data, Subjective Study Questionnaire	Patient satisfaction (increased)
Mottl-Santiago et al,^18^ 2023	Mean age, 25 y; race and ethnicity, 35% Black in doula and control groups, 47% Hispanic in doula group and 49% in control group; 75% non-US born; mean GA, 19 wk	CD,^a^ LBW, assisted vaginal delivery, postpartum hemorrhage, preterm birth, maternal depression, epidural, 5-min Apgar score, breastfeeding initiation (increased),^b^ breastfeeding duration, breastfeeding exclusivity, gestational hypertension	Immediately after birth and approximately 12 wk post partum	Edinburgh Postnatal Depression Scale	NA
Schytt et al,^29^ 2022	Mean age, 30 y; language, 37%-42% Arabic, 23% Somali, 10% Russian, 23%-26% Tigrinya, 1%-3% Polish; marital status, 23% single	Patient satisfaction,^a^ maternal depressive symptoms,^a^ episiotomy, perineal injury, postpartum hemorrhage, vacuum extraction, epidural	Before labor and 6-8 wk post partum	Edinburgh Postnatal Depression Scale; Migrant Friendly Maternity Care Questionnaire	Patient satisfaction
Shahbazi Sighaldeh et al,^37^ 2023	Ages 23-30 y (52%-60%); educational level, 42%-82% with diploma; nulliparous, 52%-78%	Labor length (decreased),^a^^,^^b^ maternal anxiety (decreased),^a^^,^^b^ pain	Anxiety evaluated at dilation 3-4 and 8-10 cm; pain at admission and hourly until delivery; satisfaction 24 h post partum	State-Trait Anxiety Inventory; Pain Visual Analog Scale; Birth Satisfaction Scale	Patient satisfaction
Wilson et al,^30^ 2017	Mean (SD) age, 30.9 (7.5) y in doula group vs 28.5 (5.7) y in control group; race and ethnicity, 6% Asian or Indian, 40% Black, 54% White in doula group vs 12% Asian and Indian, 54% Black, and 34% White in control group	Pain,^a^ empowerment feelings, coping feelings, emotional state changes	30 min after the procedure	Pain Visual Analog Score; 10-item Emotional State Assessment; 28-item Empowerment Score; Satisfaction on Likert scale of 1-10	Patient satisfaction
Zhang et al,^31^ 2020	Maternal age, 18-34 y 89.9% in intervention group and 90.1% in control group; ≥35 y approximately 10%; risk level of pregnancy, 51% low risk and 49% high risk in both groups	CD,^a^, AROM	Immediately after birth	NA: medical record review only	NA
Zhang et al,^36^ 2018	Mean age, 28 y; primigravid, 85%-96%; mean GA, 39 wk; educational level, 86%-90% attended university	Maternal depressive symptoms (decreased),^a^^,^^b^ pain (increased, compared with epidural with doula as control)^b^	PPD, 3 d and 2-4 wk post partum; pain before and 30, 60, and 120 min after analgesia	Edinburgh Postnatal Depression Scale; Pain Visual Analog Scale	NA
Ravangard et al,^35^ 2017	Age range, 16-44 y; 25%-26% of participants aged 16-26 y; educational level, 19%-21% diploma or higher degrees	Pain (decreased),^b^ maternal anxiety (decreased)^b^	Anxiety, before and after labor; pain in active phase of labor	State-Trait Anxiety Inventory; McGill Pain Questionnaire	NA
Trueba et al,^32^ 2000	Specific demographic data not reported	CD (decreased),^b^ oxytocin (decreased),^b^ epidural, labor length	Immediately after birth	NA: medical record review only	NA
Burris et al,^19^ 2025	Mean age, 28 y in doula group and 31 y in control group; insurance, 80% public in doula group and 88% in control group; race and ethnicity, 76% Black, 14% Hispanic, 5% White, 5% multiracial	Median time to first postpartum visit (decreased),^a^^,^^b^ receipt of blood pressure measuring, contraception counseling, and depression screening (increased)^a^^,^^b^	12 wk Post partum	NA: medical record review only	NA

Abbreviations: AROM, artificial rupture of membranes; CD, cesarean delivery; GA, gestational age; LBW, low birth weight; NA, not applicable; PHQ-9, 9-item Patient Health Questionnaire; PPD, postpartum depression.

^a^ Indicates article described power calculation for designated outcome.

^b^ Significant difference found between doula and control groups.

#### Birth Outcomes

Of the 22 included articles, only 13 provided a sample size calculation to justify their primary outcome.^18,19,20,21,22,26,27,28,29,30,31,36,37^ Among those focused on birth outcomes, 5 were powered to detect differences in cesarean delivery rates,^18,21,26,28,31^ of which 2 showed significant reductions in the doula group^26,28^ and the other 3 found no difference.^18,21,31^ Six additional articles reported on cesarean delivery secondarily or without linking it to sample size calculations; among these, 3 reported decreased cesarean delivery rates in the doula group^20,32,38^ and 3 found no significant difference.^25,33,39^ One study powered for labor length found significantly reduced labor times in the doula group.^37^ Six other reports^20,21,26,32,33,38^ assessed labor length secondarily, with 4 reporting significantly shorter labors among participants with doula support.^20,21,26,38^

Other birth outcomes were reported in several studies but were not designated as primary outcomes, and most showed no significant effects of doula support. Specifically, studies evaluating assisted vaginal delivery,^18,31^ episiotomy,^29,31,33^ perineal injury,^29,33^ preterm birth,^18,25^ and postpartum hemorrhage^18,26,29^ generally reported no differences between groups, except 1 study that found reduced postpartum hemorrhage in the doula group.^26^ Among 8 studies assessing epidural use,^18,21,25,28,29,32,36,38^ 2 reported significantly decreased use in the doula group.^25,28^ One study found lower oxytocin use,^32^ while another found higher rates of oxytocin that was attributed to higher rates of vaginal births.^38^ Of 5 studies assessing Apgar scores,^18,21,26,33,38^ 2 found significantly higher scores in the doula group.^21,26^ One study powered to evaluate postpartum doula support among mothers with infants in the neonatal intensive care unit found that participants experienced earlier postpartum follow-up and were less likely to miss postpartum blood pressure checks, contraception counseling, and depression screening compared with controls.^19^

#### Mental Health Outcomes

Twelve studies reported mental health outcomes,^18,19,20,25,26,27,29,34,35,36,37,38^ 5 of which were powered as the primary outcome.^20,27,29,36,37^ Seven studies assessed anxiety symptoms during labor and delivery^20,22,26,27,35,37,38^; 5 reported significantly lower anxiety levels in participants who received doula support,^20,26,27,35,37^ and 3 of those had designated anxiety as the primary outcome.^20,27,37^ Two studies identified PPD as the primary outcome: one found a significant benefit of doula support,^36^ while the other found no difference between groups.^29^ Of the remaining 4 studies measuring PPD, 3 found no significant differences^18,25,38^ and 1 pilot trial^34^ reported higher depression rates in the doula group—an outcome authors attributed to greater baseline mental health needs in the intervention group.

#### Child Development and Parenting Outcomes

Child development and parenting outcomes were assessed in 5 articles.^18,23,24,25,39^ Hans et al^25^ did not specify a primary outcome but found that doula-supported mothers were more likely to practice safe infant care. Edwards et al^23^ identified breastfeeding initiation as the primary outcome and reported significantly higher initiation in the doula group, with no difference in duration. Three additional studies also reported increased breastfeeding initiation as a secondary outcome.^18,25,39^ The study by Edwards et al^23^ specifically provided breastfeeding training to doulas, while the other 3 studies did not. Hans et al^24^ identified parenting behavior as the primary outcome and reported improved maternal interactions and lower parenting stress at 4 and 12 months but not at 24 months.

#### Pain Outcomes

Self-reported pain was assessed in 7 studies.^22,25,26,30,36,37,38^ The 2 studies during abortion procedures were powered for pain as a primary outcome, with both finding no significant difference between groups.^22,30^ Among the remaining 5 studies, 2 found no significant differences in reported pain,^37,38^ 2 observed decreased pain in the doula group,^26,35^ and 1 study reported increased pain among participants supported by a doula when compared with receiving epidural anesthesia.^36^

#### Implementation Outcomes

Implementation-related outcomes were reported in 8 studies (38.1%). ^26,28,29,30,33,34,37,38^ Six studies assessed patient satisfaction and consistently found that doula support was viewed as acceptable and desirable.^28,29,30,34,37,38^ Luo et al^26^ also reported high satisfaction among both patients and nurses. Only 1 study explicitly described intervention fidelity measures.^33^

## Discussion

This systematic review is the first, to our knowledge, to synthesize findings from clinical trials evaluating the health impacts of doula care. There was substantial heterogeneity across studies in relation to study designs, evaluated outcomes, and role of doulas, and the overall the quality of evidence was inconsistent, with many studies being underpowered or lacking rigorous design. Evidence for the effectiveness of doula care varied across outcomes, possibly as a result of these methodologic limitations. Labor-related outcomes, such as fewer cesarean deliveries and lower levels of pain, showed limited or inconsistent associations, whereas outcomes related to increased breastfeeding initiation and improved maternal anxiety demonstrated more consistent associations with doula care.

The findings related to perinatal mental health are promising, given the profound role that perinatal mental health plays in maternal well-bring and child outcomes. Caregivers’ experiences of depression and anxiety can disrupt bonding, impair parenting, and negatively affect long-term development.^40,41,42,43^ Notably, across included trials, mental health benefits associated with doula care were more consistently observed for anxiety-related symptoms, whereas depressive symptoms were less consistent. This pattern may reflect differences in study quality and timing, as all studies assessing anxiety focused on the intrapartum period, when doula support is most intensive. Depressive symptoms are often multifactorial and develop over longer time spans than most of the included studies addressed,^44^ which highlights an important area for future research. Current literature and clinical guidelines emphasize the importance of addressing mental health across the entire perinatal experience and recommend a range of treatments, including psychotherapy and pharmacologic management.^45,46^ As an adjunct to these approaches, doulas may provide a distinctive, nonpharmacologic layer of emotional and practical support for expectant parents, and our review highlights emerging evidence from clinical trials that suggests their potential to enhance perinatal mental health outcomes.

While the evidence we found for mental health was more consistent, findings for other domains such as mode of delivery, pain, and birth-related complications were more variable. It is important to contextualize the mixed nature of our findings within the broader literature, which includes observational and retrospective studies. Numerous cohort studies have demonstrated associations between doula support and reduced rates of cesarean delivery.^13,14,15,16^ Other demonstrated associations include improvements in outcomes such as preterm birth^47^ and reduced rates of labor induction.^48^ Similarly, systematic reviews not limited to clinical trials have found consistent and convincing evidence supporting the benefits of doula care.^49,50^ A 2017 Cochrane meta-analysis also reported positive effects, including increased rates of spontaneous vaginal birth, shorter labor duration, and reduced cesarean delivery rates; however, the quality of evidence was rated as low, and relative risks ranged from 0.75 to 1.08.^51^

The discrepancy between findings from clinical trials and other types of research raises important questions. One explanation may be methodologic: few trials were adequately powered to detect significant differences in primary outcomes, and designs varied widely in how doula care was implemented. Most studies did not describe doula recruitment or training, and only one reported average contact hours.^34^ A notable gap was the lack of attention to crossover, as control participants may have independently accessed doula care, especially in the context of increased Medicaid coverage and community availability.^10,52^ Such unmeasured exposure may threaten internal validity and obscure true effects. Without standardized delivery or sufficient detail on intervention fidelity, evaluating doulas’ impact through clinical trials remains difficult. State-level variation in Medicaid expansion and doula reimbursement policies may also partially explain heterogeneity in access and outcomes across studies, complicating comparisons and limiting generalizability.^11^ Greater investment in rigorous, well-designed trials with consistent reporting standards is essential to establish efficacy and inform policies expanding doula coverage for underserved communities disproportionately affected by barriers to care^53,54^ and adverse birth outcomes.^4,5^

In addition to strengthening the evidence base, future research must address critical gaps in equity and representation. Few studies tailored doula care to marginalized populations, and none addressed the needs of individuals in the carceral system or mothers of children with complex medical needs—groups often lacking social support. These omissions are critical, as doulas provide emotional, informational, and advocacy support that may be especially beneficial for those facing stress, trauma, or systemic disadvantage. Existing evidence suggests that doulas could be impactful in these contexts: caregivers of children with medically complex conditions reported lower depression and anxiety when supported in navigating the health care system,^55^ and incarcerated pregnant individuals describe doula support as both beneficial and highly desired.^56,57^ Intimate partner violence, which affects as many as 1 in 4 pregnant people,^58,59^ was also notably absent from the existing literature despite pregnancy being a time of increased risk and need for support. Future research endeavors should aim to center these populations to ensure doula care advances equity for those most vulnerable.

Given that doula care is often associated with childbirth, it is unsurprising that most studies focused on intrapartum support. However, full-spectrum doula care spans the prenatal, birthing, and postpartum periods. Only 7 studies included postpartum care, despite its importance for fostering maternal-infant bonding,^42^ promoting parental competence,^60^ and maintaining mental health.^41,61^ These areas are vital for long-term maternal and infant well-being. Furthermore, doulas are increasingly involved in a wider range of reproductive health contexts, such as bereavement,^62,63^ abortion,^64^ and infertility,^65^ as well as nonperinatal care (eg, end of life), where emotional and psychologic support is critical. Only 2 included studies^22,30^ addressed abortion-related care, and no trials have evaluated doula care for infertility, pregnancy loss, or nonreproductive care. Expanding research beyond labor and delivery is essential to fully capture the scope and impact of doula support across the reproductive health continuum.

A final key finding of this review is the limited attention to implementation science, or the study of methods to increase the uptake and sustainment of innovative practices.^66^ Only 8 studies^26,28,29,30,33,34,37,38^ examined an implementation outcome, most commonly patient satisfaction. These studies consistently demonstrated that doulas are acceptable to and desired by patients, aligning with wider doula literature and underscoring the importance of this source of support.^9,67^ No studies were described as implementation or hybrid trials,^68^ signaling a clear need for examining implementation determinants, strategies, or outcomes. Additionally, no studies explicitly examined how insurance coverage or Medicaid reimbursement influenced implementation or uptake of doula services. These gaps severely limit what is understood about how to effectively implement doula care. Future studies should explore barriers and facilitators to doula care, develop testable implementation strategies (eg, approaches to training and integration into the health care system), and evaluate the impact of implementation strategies on a broader range of implementation outcomes. Evidence from such trials will be critical to inform reimbursement policies and ensure equitable access as demand for doula services continues to grow.

Clinically, these findings underscore the opportunity to recognize and expand the role of doulas across the care continuum, from prenatal and labor support to postpartum, abortion, bereavement, and fertility contexts, as well as nonreproductive settings such as end of life. Integrating doulas into multidisciplinary maternity and reproductive health teams may enhance patient-centered care, improve communication between patients and clinicians, and address psychosocial needs that traditional models often overlook. Such integration requires consistent training standards, clear role delineation, and institutional support to ensure equitable access and effective collaboration.

At the policy level, stronger evidence is necessary to guide equitable Medicaid reimbursement, training standards, and sustainable workforce integration. Implementation research linking program structure to measurable outcomes can inform scalable, equitable models of doula care. Collectively, rigorous, equity-focused research and supportive clinical and policy frameworks are vital to realizing the full potential of doula care in addressing persistent maternal-infant health inequities.

### Limitations

This review has some limitations. Despite a broad search strategy, it is possible that relevant studies were missed due to inconsistent terminology or publication bias. We included studies conducted outside the US but published in English, which may introduce heterogeneity related to cultural context, health system differences, or publication standards. Finally, there was wide variation in sample sizes and study design quality, which may introduce bias and limit the generalizability of findings.

## Conclusions

This systematic review found that doula support was most consistently associated with improved maternal anxiety and breastfeeding initiation, with emerging evidence for improving use of postpartum follow-up. Findings for other outcomes (eg, cesarean delivery, depression) were mixed. These results contrast with observational research that has more consistently demonstrated benefits across a broader range of maternal and infant outcomes, suggesting that existing clinical trials may be underpowered or methodologically limited. More robust study designs, including standardized outcome measures, preregistered protocols, and adequate statistical power, are essential to strengthen causal inference and clarify the true impact of doula care. Future longitudinal clinical trials that span the entirety of the perinatal period are needed to assess when doula care can be most impactful. Standardizing the core components of doula care and clearly reporting on their implementation would facilitate comparisons across studies and promote evidence-based implementation.

## Notes

Supplement 1. **eTable.** Evidence Quality Assessment via Oxford Centre for Evidence-Based Medicine
eReferences

Supplement 2.
Data Sharing Statement
