# Supplementary material for: Doula Care and Health Outcomes: A Systematic Review
Source: JAMA Netw Open. 2026 Apr 21;9(4):e268416. doi: 10.1001/jamanetworkopen.2026.8416 (PMC13100869; doi:10.1001/jamanetworkopen.2026.8416)
Supplement: Supplement 1. — eTable. Evidence Quality Assessment via Oxford Centre for Evidence-Based Medicine eReferences [file jamanetwopen-e268416-s001.pdf]

Supplementary Online Content

Groves P, Williams H, Salter CL, et al. Doula care and health outcomes: a systematic review. *JAMA Netw Open.* 2026;9(4):e268416. doi:10.1001/jamanetworkopen.2026.8416

eTable. Evidence Quality Assessment via Oxford Centre for Evidence-Based Medicine
eReferences

This supplementary material has been provided by the authors to give readers additional information about their work.

© 2026 Groves P et al. *JAMA Network Open*.

eTable. Evidence Quality Assessment via Oxford Centre for Evidence-Based Medicine

Level of Evidence Quality	Studies, No.
Level 1: Properly powered and conducted randomized clinical trial	11 studies[1-11]
Level 2: Well-designed controlled trial without randomization; prospective comparative cohort trial	11 studies[12-22]

eReferences

1. Akbarzadeh M, Masoudi Z, Zare N, Vaziri F. Comparison of the effects of doula supportive care and acupressure at the BL32 point on the mother's anxiety level and delivery outcome. Iran J Nurs Midwifery Res. 2015;20(2):239-246. Medline:25878703
2. Campbell DA, Lake MF, Falk M, Backstrand JR. A randomized control trial of continuous support in labor by a lay doula. J Obstet Gynecol Neonatal Nurs. 2006;35(4):456-464. Medline:16881989 doi:10.1111/j.1552-6909.2006.00067.x
3. Chor J, Hill B, Martins S, Mistretta S, Patel A, Gilliam M. Doula support during first-trimester surgical abortion: a randomized controlled trial. Am J Obstet Gynecol. 2015;212(1):45.e1-45.e6. doi:10.1016/j.ajog.2014.06.052
4. Luo Q, Xu B, Qian M, Lu Z. Effects of integrating nursing early warning systems with doula-assisted childbirth on natural childbirth rates and neonatal outcomes: a single center prospective study. Altern Ther Health Med. 2025;31(3):100-106. Medline:38814602
5. Masoudi Z, Kasraeian M, Akbarzadeh M. Assessment of educational intervention and acupressure during labor on the mother's anxiety level and arterial oxygen pressure of the umbilical cord of infants (PO2): a randomized controlled clinical Trial. J Educ Health Promot. 2022;11:86. Medline:35573609 doi:10.4103/jehp.jehp_685_20
6. McGrath SK, Kennell JH. A randomized controlled trial of continuous labor support for middle-class couples: effect on cesarean delivery rates. Birth. 2008;35(2):92-97. Medline:18507579 doi:10.1111/j.1523-536X.2008.00221.x
7. Mottl-Santiago J, Dukhovny D, Cabral H, et al. Effectiveness of an enhanced community doula intervention in a safety net setting: a randomized controlled trial. Health Equity. 2023;7(1):466-476. Medline:37731785 doi:10.1089/heq.2022.0200
8. Schytt E, Wahlberg A, Eltayb A, Tsekhmestruk N, Small R, Lindgren H. Community-based bilingual doula support during labour and birth to improve migrant women's intrapartum care experiences and emotional well-being: findings from a randomised controlled trial in Stockholm, Sweden [NCT03461640]. PLoS One. 2022;17(11):e0277533. Medline:36399476 doi:10.1371/journal.pone.0277533
9. Wilson SF, Gurney EP, Sammel MD, Schreiber CA. Doulas for surgical management of miscarriage and abortion: a randomized controlled trial. Am J Obstet Gynecol. 2017;216(1):44.e1-44.e6. doi:10.1016/j.ajog.2016.08.039

© 2026 Groves P et al. *JAMA Network Open*.

10. Zhang L, Zhang L, Li M, et al. A cluster-randomized field trial to reduce cesarean section rates with a multifaceted intervention in Shanghai, China. BMC Med. 2020;18(1):27. Medline:32054535 doi:10.1186/s12916-020-1491-6
11. Burris HH, Darden N, Power M, et al. Postpartum care in the neonatal intensive care unit, PeliCaN: a randomized controlled trial. Am J Obstet Gynecol MFM. 2025;7(7):101689. Medline:40334984 doi:10.1016/j.ajogmf.2025.101689
12. Chen CC, Lee JF. Effectiveness of the doula program in Northern Taiwan. Tzu Chi Med J. 2020;32(4):373-379. Medline:33163384 doi:10.1142/S0192415X20500196
13. de Moraes EDV, Ribeiro MS, Erbert C, Prado CAC, Moises ECD. Impact of doula's continuous support on serotonin release in parturients: a pilot randomized clinical trial. Rev Bras Ginecol Obstet. Published online April 29, 2024. doi:10.61622/rbgo/2024rbgo27
14. Edwards RC, Thullen MJ, Korfmacher J, Lantos JD, Henson LG, Hans SL. Breastfeeding and complementary food: randomized trial of community doula home visiting. Pediatrics. 2013;132(suppl 2):S160-S166. Medline:24187119 doi:10.1542/peds.2013-1021P
15. Gjerdingen DK, McGovern P, Pratt R, Johnson L, Crow S. Postpartum doula and peer telephone support for postpartum depression: a pilot randomized controlled trial. J Prim Care Community Health. 2013;4(1):36-43. Medline:23799688 doi:10.1177/2150131912451598
16. Gruber KJ, Cupito SH, Dobson CF. Impact of doulas on healthy birth outcomes. J Perinat Educ. 2013;22(1):49-58. Medline:24381478 doi:10.1891/1058-1243.22.1.49
17. Hans SL, Thullen M, Henson LG, Lee H, Edwards RC, Bernstein VJ. Promoting positive mother–infant relationships: a randomized trial of community doula support for young mothers. Infant Ment Health J. 2013;34(5):446-457. doi:10.1002/imhj.21400
18. Hans SL, Edwards RC, Zhang Y. Randomized controlled trial of doula-home-visiting services: impact on maternal and infant health. Matern Child Health J. 2018;22(suppl 1):105-113. Medline:29855838 doi:10.1007/s10995-018-2537-7
19. Shahbazi Sighaldeh S, Azadpour A, Vakilian K, Rahimi Foroushani A, Vasegh Rahimparvar SF, Hantoushzadeh S. Comparison of maternal outcomes in caring by doula, trained lay companion and routine midwifery care. BMC Pregnancy Childbirth. 2023;23(1):765. Medline:37907873 doi:10.1186/s12884-023-05987-7
20. Zhang Y, Johnston L, Ma D, Wang F, Zheng X, Xu X. An exploratory study of the effect of labor pain management on postpartum depression among Chinese women. Ginekol Pol. 2018;89(11):627-636. Medline:30508215 doi:10.5603/GP.a2018.0107
21. Ravangard R, Basiri A, Sajjadnia Z, Shokrpour N. Comparison of the effects of using physiological methods and accompanying a doula in deliveries on nulliparous women's anxiety and pain: a case study in Iran. Health Care Manag (Frederick). 2017;36(4):372-379. Medline:28961642 doi:10.1097/HCM.0000000000000188
22. Trueba G, Contreras C, Velazco MT, Lara EG, Martínez HB. Alternative strategy to decrease cesarean section: support by doulas during labor. J Perinat Educ. 2000;9(2):8-13. Medline:17273201 doi:10.1624/105812400X87608

© 2026 Groves P et al. *JAMA Network Open*.
