# Supplementary material for: Doula Care and Health Outcomes: A Systematic Review
Source: JAMA Netw Open. 2026 Apr 21;9(4):e268416. doi: 10.1001/jamanetworkopen.2026.8416 (PMC13100869; doi:10.1001/jamanetworkopen.2026.8416)
Supplement: Supplement 2. — Data Sharing Statement [file jamanetwopen-e268416-s002.pdf]

Data Sharing Statement

Groves. Doula Care and Health Outcomes. *JAMA Netw Open*. Published April 21, 2026.
doi:10.1001/jamanetworkopen.2026.8416

Data
Data available: No

Additional Information
Explanation for why data not available: The data is already available given this is a review of previously published articles.
